# The effect of antiretroviral treatment on selected genes in whole blood from HIV-infected adults sensitised by *Mycobacterium tuberculosis*

**DOI:** 10.1371/journal.pone.0209516

**Published:** 2018-12-27

**Authors:** Nishtha Jhilmeet, David M. Lowe, Catherine Riou, Thomas J. Scriba, Anna Coussens, Rene Goliath, Robert J. Wilkinson, Katalin Andrea Wilkinson

**Affiliations:** 1 Wellcome Centre for Infectious Diseases Research in Africa, Institute of Infectious Disease and Molecular Medicine, University of Cape Town, Cape Town, South Africa; 2 South African Tuberculosis Vaccine Initiative, Institute of Infectious Disease and Molecular Medicine, University of Cape Town, Cape Town, South Africa; 3 Department of Pathology, University of Cape Town, Cape Town, South Africa; 4 Department of Medicine, University of Cape Town, Cape Town, South Africa; 5 The Francis Crick Institute, London, United Kingdom; 6 Department of Medicine, Imperial College London, London, United Kingdom; Institut de Pharmacologie et de Biologie Structurale, FRANCE

## Abstract

HIV-1 co-infection is a leading cause of susceptibility to tuberculosis (TB), with the risk of TB being increased at all stages of HIV-1 infection. Antiretroviral treatment (ART) is the most effective way to reduce the risk of TB in HIV-1 co-infected people. Studying protective, ART-induced, immune restoration in HIV-1 infected individuals sensitised by *Mycobacterium tuberculosis* (*Mtb*) can thus help identify mechanisms of protection against TB. In order to understand ART-mediated prevention of TB in HIV-1 infected adults, we investigated the expression of 30 genes in whole blood from HIV-1 infected patients during the first 6 months of ART-induced immune reconstitution. The 30 selected genes were previously described to be differentially expressed between sorted *Mtb* specific central and effector memory CD4 T cells. HIV-1 infected persons sensitised by *Mtb* were recruited in Khayelitsha, South Africa, when initiating ART. RNA was extracted from whole blood at initiation and 1, 3 and 6 months of ART. qRT-PCR was used to determine gene expression and three reference ‘housekeeping’ genes were used to calculate the fold change in the expression of each gene relative to day 0 of ART. Results were assessed longitudinally. We observed a decrease in the expression of a number of genes at 6 months of ART, reflecting a decrease in immune activation. However, following correction for multiple comparisons and increasing CD4 counts, only the decrease in *CD27* gene expression remained statistically significant. While not statistically significant, a number of genes also showed increased expression at various timepoints, illustrating the broad regeneration of the T cell pool in HIV-1 infected adults on ART. Our findings generate hypotheses underlying ART- induced protective immune reconstitution and may pave the way for future studies to evaluate ART mediated prevention of TB in HIV-1 infected persons.

## Introduction

Tuberculosis (TB) is the leading bacterial cause of death worldwide [1] and HIV infected persons are 20–30 times more likely to develop TB than HIV uninfected persons, who have a 5–10% chance of developing active TB in their lifetime [2]. HIV-1 co-infection is the leading cause of susceptibility to TB, and antiretroviral treatment (ART) is the most effective way to reduce the risk of TB in HIV-1 co-infected persons, reducing tuberculosis incidence by up to 67% [3]. In order to identify mechanisms of ART-mediated prevention of TB in HIV-1 infected persons, we longitudinally analysed a group of HIV-1 infected persons starting ART. Our hypothesis was that this highly susceptible group, who undergo immune restoration through ART and thereby become less susceptible to TB, will yield insight into understanding protective mechanisms against human TB. A previous longitudinal follow-up of 19 HIV infected adults with *Mycobacterium tuberculosis* (*Mtb*) sensitisation over 48 weeks of ART showed that the strongest correlate of increased ART mediated immunity was the expansion of the less differentiated central memory CD4 T cell pool [4].

The importance of central memory T cells (TCM) in protection against TB has been highlighted by studies addressing the mechanisms of action of promising vaccine candidates, using animal models. The subunit vaccine H1 (containing Ag85B and ESAT-6) boosts CD4^+^KLRG1^-^IL-2^-^secreting TCM [5]; the recombinant BCG Delta*ureC*::*hly* vaccine mediated protection is based on the expansion of central memory CD4 T cells that are CXCR5^+^CCR7^+^ and express low levels of the transcription factors T-bet and Bcl-6 [6] the sustained protection induced by the H56/CAF01 subunit vaccine was mediated by the less differentiated lung parenchyma homing CD4 T cells that express low levels of KLRG1 and secrete high amounts of IL-2 and IL-17A [7]. In humans, *Mtb* infected adolescents were followed longitudinally in order to understand the mechanisms underlying progression from infection to pulmonary TB disease. Those who progressed from latent infection to active TB disease displayed T cell activation (indicated by elevated expression of HLA-DR on CD4 T cells) and a decrease in relative proportions of CD45RA^-^CCR7^+^ central memory CD4 and CD8 T cells [8]. More recently, Chiacchio et al found that antiretroviral and anti-tuberculosis therapies significantly increased the frequency of Mtb specific CD4 T cells in HIV-TB co-infected patients with an increase in the central memory compartment [9]. Overall, these findings support the role of central memory CD4 T cells as potential correlates of protection in TB.

To broadly investigate the ART-induced reconstitution of the T cell pool in HIV-1 infected persons during the first 6 months of ART, we performed quantitative RT-PCR using RNA extracted from whole blood, for genes selected on the basis of differential expression between sorted *Mtb* specific TCM and effector memory (TEM) CD4 T cells as described [10]. Thus *ICOS*, *SELL*, *PRKCA*, *TCF7L*, *LEF1*, *NFKB1*, *CD38*, *ITK*, *IGF1R*, *ARHGEF18*, *AXIN2*, *CCR7* and *CD27* had a significantly higher expression in sorted *Mtb* specific TCM compared to TEM while the genes *GNLY*, *RORC*, *TGFB1*, *PRF1*, *CCL5*, *IFNG*, *CCR2*, *GATA*, *GZMA*, *GZMB*, *GZMK*, *PRR5L*, *TXB21*, *IL2RB*, *CCR4*, *CCR5* and *FAM129A* had a significantly higher expression in sorted *Mtb* specific TEM compared to TCM. We evaluated the change in gene expression during the first 6 months of ART and hypothesised that the transcription of central memory T cell associated genes would increase over time in HIV infected persons during the first 6 months of ART.

## Materials and methods

### Study population and sample collection

Ethical approval for the study was obtained from the University of Cape Town Faculty of Health Sciences Human Research Ethics Committee (HREC 245/2009 and 545/2010). All participants gave written informed consent in accordance with the Declaration of Helsinki. HIV infected persons starting ART were recruited from the Ubuntu Clinic in Khayelitsha, South Africa, during two longitudinal studies in 2011–2012. The two cohorts have previously been described [11, 12]. Measurement of CD4 count and HIV viral load was performed by the South African National Health Laboratory Service by flow cytometry and polymerase chain reaction respectively. Blood for RNA extraction was collected in Tempus tubes at baseline and after one (1M), three (3M) and six months (6M) of receiving ART, and stored for future use. Additional blood was collected to establish *Mtb* sensitisation using the Quantiferon Gold In-tube (QFT) assay and an in-house Enzyme-Linked ImmunoSpot assay (ELISpot) as described [12].

### RNA Isolation and characterisation

Tubes were stored at -20°C until batched processing using the Tempus Spin RNA Isolation kit (Thermofisher) according to the manufacturer’s recommendations. RNA quantity and quality (integrity) were assessed by a Nanodrop 2000c spectrophotometer (Thermo Scientific) and an Agilent RNA 6000 Nano Kit on the Agilent 2100 Bioanalyzer. RNA was reverse transcribed to cDNA using the SuperScript III First-Strand Synthesis System for RT-PCR, using 9μL RNA in a total reaction volume of 20μL per sample in a Bio-Rad thermocycler at 50°C for 50 minutes followed by termination at 85°C for 5 minutes and a hold at 4°C. cDNA was stored at -20°C until use.

### RT-PCR using TaqMan Fast Advance Mastermix and probes

Thirty memory T cell specific genes were selected for analysis based on significantly elevated expression in sorted *Mtb*-specific TCM (13 genes) and TEM (17 genes). The selection of genes was based on results from a previous study [10], comparing the expression of 96 genes measured by microfluidic RT-PCR in *Mtb*-specific CD4+ T cells identified by HLA class II tetramers, sorted based on the surface expression of CD45RA, CCR7 and CD27, and classified into naïve, stem cell like memory, central memory and effector memory phenotype. We selected the genes that were differentially expressed between the sorted *Mtb* specific central memory and effector memory T cells (S1 Table). Based on a recent systematic review of endogenous controls in gene expression studies [13], we included three reference genes, *18S*, *ACTB* and *GAPDH*, in order to increase the stringency of the analysis. TaqMan probes used for RT-PCR are summarised in S1 Table. Eighteen μL mastermix containing 10μL TaqMan Fast Advance Master Mix (2x), 1 μL TaqMan PDAR and 7μL nuclease-free water, was added to all wells in a 96-well optical reaction plate, with each well containing 2μL of sample cDNA. The plate was run on a QuantStudio 7 Flex (Applied Biosystems) in fast mode, for 40 cycles, as follows: polymerase activation at 95°C for 20 seconds, denaturation at 95°C for 1 second, extension at 60°C for 20 seconds. Quantstudio 6 and 7 Flex Real-Time PCR software (Applied Biosystems) was used for the instrument setup. This software measures the PCR cycle at which the gene of interest begins to exponentially expand above the threshold limit, referred to as the Ct value.

### Data analysis

**Fig 1:**
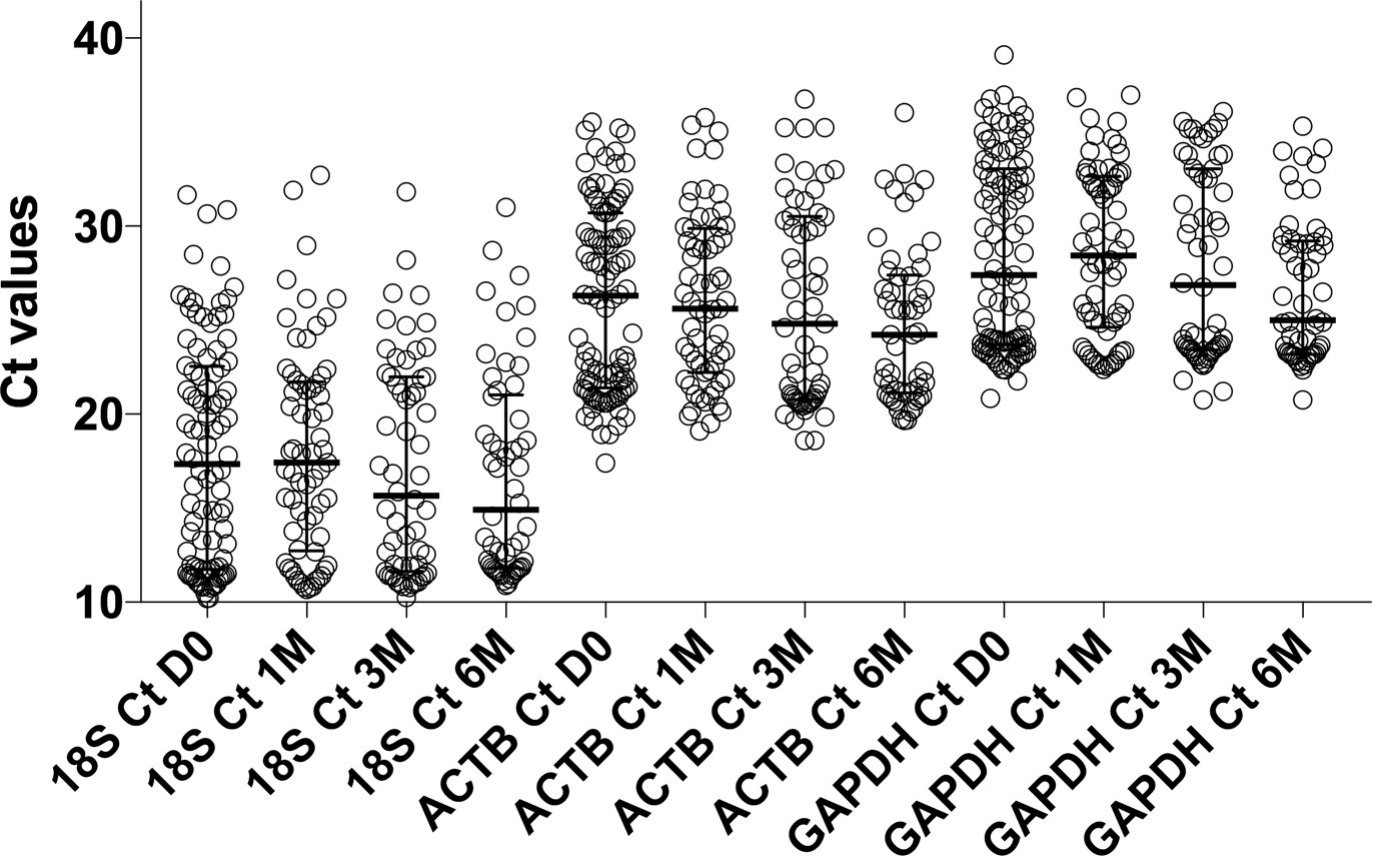
Ct values of the reference genes *18S*, *ACTB* and *GAPDH* indicated no significant change in the median and IQR at all timepoints.

Ct values were normalised to the reference genes *18S*, *ACTB* and *GAPDH*. Inspection of the raw Ct values for the three reference genes indicated no significant change in the median and IQR at all timepoints (Fig 1). Based on this, we normalised our gene of interest Ct values to the average expression of the three reference genes at each timepoint. Thus, Delta Ct was calculated using the formula: Delta Ct = Ct (gene of interest) at timepoint—Ct (average of reference genes) at timepoint, timepoint meaning day 0, 1M, 3M, 6M respectively. Transcripts that failed to amplify during the 40 cycles from all samples, were excluded from analysis. Next, we calculated the fold change in the expression of each gene relative to day 0 of ART, using the formula: Fold Change = 2^ [(Delta Ct at timepoint)—(Delta Ct at D0)]. In order to account for the fact that we used bulk RNA extracted from whole blood to evaluate T cell specific genes, and that the CD4 T cell counts increased over time due to ART (Table 1), we further calculated the CD4 count corrected fold change using the formula: Corrected Fold change (at timepoint) = Fold Change / [(CD4 at timepoint / CD4 at D0)]. Increased gene expression is indicated by fold change values of > 1.

**Table 1:** Effect of ART on CD4 counts and Viral loads of patients longitudinally, and sensitisation to *Mtb* as determined by interferon gamma release assays.

Median (IQR)	HIV Infected Persons on ART (n = 48)			
Time on ART	Day 0	1M	3M	6M
CD4 count (cells/μl blood)	199 (115–255)	275 (182–366)	301 (191–372)	320 (218–426)
Viral Load* (copies/mL blood)	79253 (40345–209 238)	316 (123–643)	40 (39–108)	40 (39–40)
QFT Gold InTube (IU/ml)	1.5 (0.51–7.3) (n = 48)	1.7 (0.21–12) (n = 41)	0.73 (0.18–6.9) (n = 38)	1.6 (0.17–4.8) (n = 34)
% positive by QFT Gold InTube	65	63	46	42
ELISpot (SFC/million PBMC)	28 (4–230) (n = 41)	72 (14–540) (n = 41)	70 (20–496) (n = 38)	72 (21–198) (n = 34)
% positive by ELISpot**	56	63	66	54

* Limit of detection is 40 and the value assigned to <40 copies/ml was 39.

** Defined as ≥ 29 SFC/million PBMC.

### Flow cytometry analysis

Cryopreserved peripheral blood mononuclear cells were thawed, counted, rested and stained for the following surface markers: CD14 (APC-cy7), CD3 (BV785), CD4 (PE-cy7), CD8 (V500), CD45RA (BV570), CD27 (BV711), CD62L (FITC), CD38 (APC) and viability dye (APC-Cy7). Following washing, cells were acquired on a LSR-II (BD) and analysed using FlowJo.

### Statistical analysis

Data was analysed using GraphPad Prism (version 6.0) for Mac. The Normality of the data was determined using the D’Agostino and Pearson normality test. Unpaired data that was not normally distributed was analysed using the Mann-Whitney U test. Correction for multiple comparisons was performed using Bonferroni correction by multiplying p values with the number of comparisons (n)-1.

## Results

**Fig 2:**
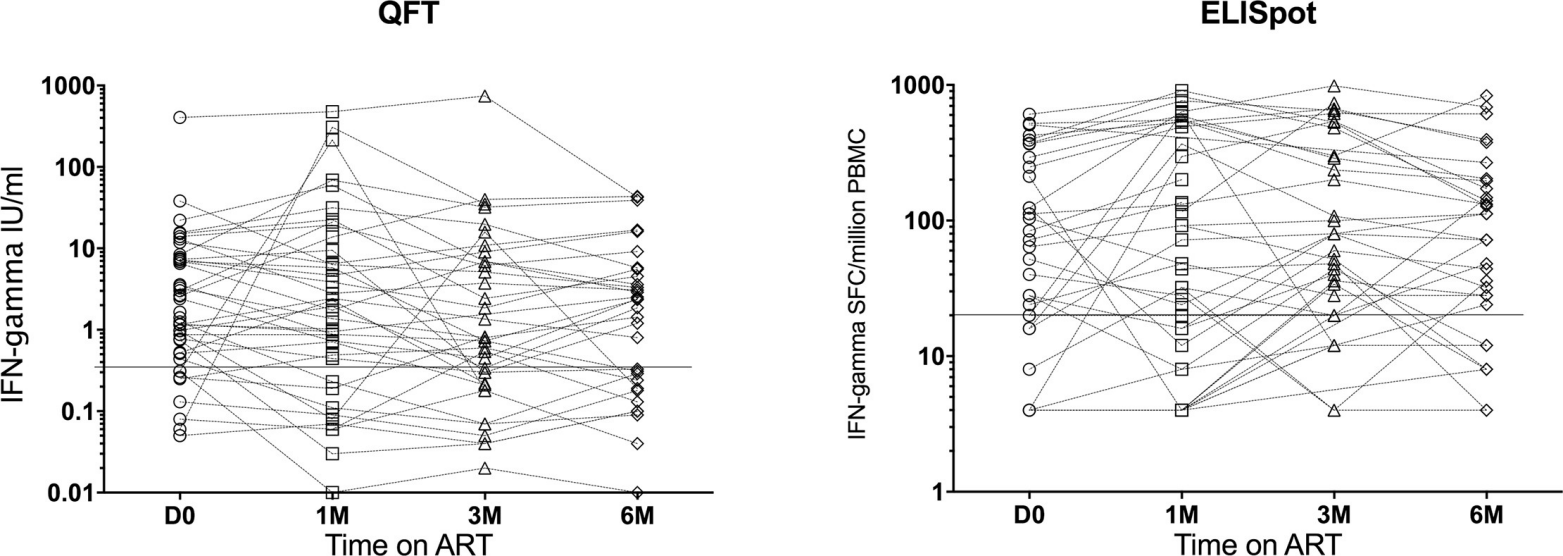
MTB sensitisation of the patients as determined by interferon gamma release assays QFT-gold in-tube (cutoff 0.35 IU/ml) and in house ELISpot (cut-off 20 spot forming cells per million PBMC). All patients were positive in at least one assay, at least one timepoint during the longitudinal follow up.

**Fig 3:**
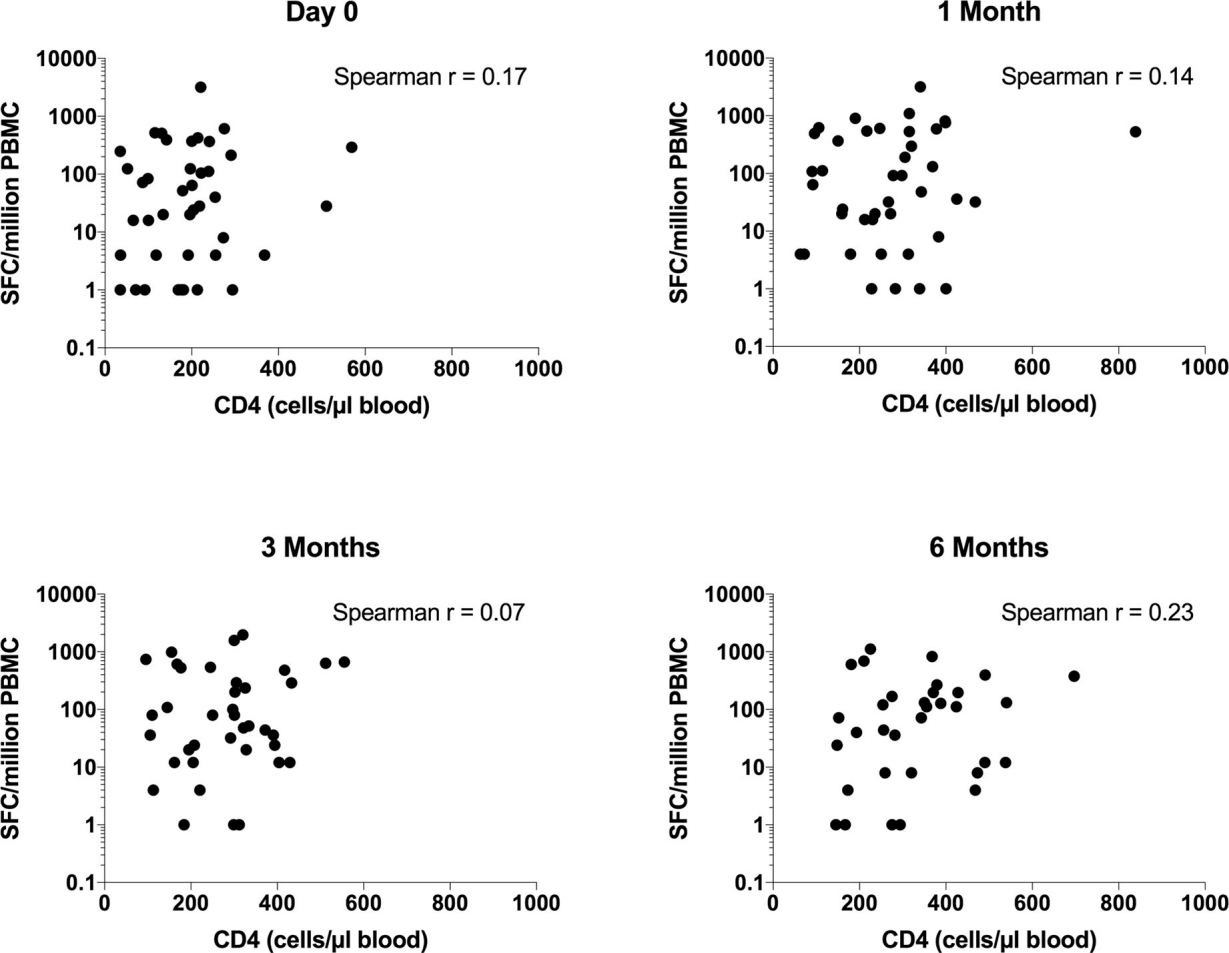
Elispot responses expressed as spot forming cells per million PBMC, related to CD4 counts in cells per microliter blood, at each timepoint. There was no correlation between the ELISpot response and the CD4 counts.

Blood stored in Tempus tubes for RNA extraction was available from n = 48 HIV infected persons (36 females, 12 males, median age 35 years, IQR 29–38) at all follow up timepoints, all of whom were sensitised by *Mtb* as determined by interferon gamma release assays QFT and ELISpot (Table 1 and Fig 2). While not all patients had samples available for testing *Mtb* sensitisation at all timepoints, all patients were positive by at least one assay on at least one timepoint during the longitudinal follow up, indicating *Mtb* sensitisation and we infer current or prior latent TB infection. The effect of ART on CD4 counts and HIV viral loads of the patients is also summarized in Table 1, showing that all patients experienced CD4 reconstitution and viral load suppression during the longitudinal follow up. Thus, the median CD4 count increased from 199 (IQR 115–255) to 320 (IQR 218–426) cells/μl blood and median HIV viral load decreased from 79253 (IQR 40345–209238) to 40 (IQR 39–40) copies/mL blood between day 0 and 6M of ART. There was no correlation between the ELISpot response and CD4 count at any of the timepoints (Fig 3).

All transcripts were normalised to the average of the three references (*18S*, *ACTB* and *GAPDH*) in order to generate Delta-Ct values. The fold change in the expression of each gene relative to day 0 of ART, was calculated and summarized in Table 2. Several genes showed an increase at various time points, including *CCL5*, *IL2RB* and *TBX21* showing an increased expression relative to day 0 at both 1M and 3M of ART. However, those showing an increase at all three time points (1M, 3M and 6M) were *CD38* and *SELL* (Table 2, fold change values highlighted in bold). A number of genes showed a significantly decreased expression at 6M of ART, such as *CD27*, *LEF1*, *CCL5*, *CCR4*, *CCR5*, *FAM129A*, *GZMK and IL2RB* (Table 2, p values in bold). Bonferroni correction for multiple comparisons resulted in none of these changes remaining statistically significant.

**Table 2:** Fold change (median and IQR) of gene expression between day 0 and 1M, 3M, 6M of ART, without correction for CD4 counts. Increased gene expression is indicated by values >1. p-values listed compare gene expression between day 0 and 6 months of ART, as well as after Bonferroni correction for multiple comparisons (p_corr_*).

Gene	1M	3M	6M	p value	p_corr_*
**TCM specific genes**					
*ARGHEF18*	**1.1 (0.59–2)**	0.97 (0.39–2.9)	0.86 (0.019–1.9)	0.164	ns
*AXIN2*	0.7 (0.15–1.5)	0.74 (0.054–1.6)	0.51 (0.078–1)	0.464	ns
*CCR7*	0.82 (0.39–1.4)	0.83 (0.41–1.5)	0.49 (0.0051–1.1)	0.094	ns
*CD27*	1 (0.69–1.7)	0.93 (0.35–1.8)	0.58 (0.0032–1.1)	**0.010**	ns
***CD38***	**1.6 (0.61–3.3)**	**1.7 (0.12–3.1)**	**1.1 (0.014–3.7)**	0.387	ns
*ICOS*	0.97 (0.37–1.6)	**1.2 (0.32–2.3)**	0.69 (0.054–2.3)	0.515	ns
*IGF1R*	0.6 (0.42–0.99)	0.62 (0.22–2.2)	0.54 (0.0059–1.4)	0.391	ns
*ITK*	1 (0.25–2.7)	**1.1 (0.15–4.2)**	0.65 (0.013–2.4)	0.308	ns
*LEF1*	0.94 (0.36–2.2)	0.91 (0.11–3.6)	0.4 (0.018–1.1)	**0.028**	ns
*NFKB1*	0.85 (0.44–1.8)	1 (0.48–2.5)	0.88 (0.0046–1.7)	0.384	ns
*PRKCA*	0.52 (0.069–5.2)	0.23 (0.01–1.6)	0.47 (0.0096–5.4)	0.617	ns
***SELL***	**1.3 (0.67–1.8)**	**1.2 (0.5–2.1)**	**1.3 (0.21–1.8)**	0.438	ns
*TCF7L2*	**1.6 (0.57–2.4)**	0.79 (0.17–3)	0.69 (0.012–3.7)	0.217	ns
**TEM specific genes**					
*CCL5*	**1.1 (0.6–1.5)**	**1.3 (0.54–2.1)**	0.63 (0.23–1.6)	**0.031**	ns
*CCR2*	0.62 (0.36–1.2)	0.77 (0.075–1.9)	0.29 (0.019–1.3)	0.120	ns
*CCR4*	0.85 (0.42–1.5)	0.46 (0.057–1.4)	0.38 (0.01–1.2)	**0.033**	ns
*CCR5*	**1.1 (0.67–1.8)**	0.77 (0.14–2)	0.3 (0.026–2.8)	**0.049**	ns
*FAM129A*	**1.1 (0.53–1.8)**	0.91 (0.47–1.7)	0.7 (0.012–1.1)	**0.015**	ns
*GATA3*	0.77 (0.36–1.8)	0.5 (0.2–1.2)	0.47 (0.0025–1.6)	0.247	ns
*GNLY*	0.69 (0.39–1.3)	0.99 (0.46–1.9)	0.63 (0.21–1.3)	0.434	ns
*GZMA*	**1.1 (0.55–1.7)**	0.94 (0.5–2.8)	0.72 (0.074–2)	0.184	ns
*GZMB*	0.94 (0.46–1.8)	**1.3 (0.29–3.1)**	0.61 (0.023–1.7)	0.109	ns
*GZMK*	0.91 (0.49–1.4)	0.85 (0.34–2.2)	0.54 (0.022–1.2)	**0.047**	ns
*IFNG*	0.86 (0.34–1.9)	**1.4 (0.29–2.8)**	0.4 (0.0037–2.4)	0.292	ns
*IL2RB*	**1.1 (0.39–1.7)**	**1.7 (0.3–3.2)**	0.54 (0.0031–1.5)	**0.038**	ns
*PRF1*	**1.5 (0.28–11)**	0.82 (0.14–8.6)	0.5 (0.019–3.1)	0.052	ns
*PRR5L*	**1.3 (0.47–2.8)**	0.86 (0.2–1.9)	0.67 (0.011–2.2)	0.099	ns
*RORC*	0.75 (0.34–2.1)	0.25 (0.0001–2.4)	0.026 (0.0008–0.62)	0.077	ns
*TBX21*	**1.2 (0.31–4.6)**	**1.1 (0.096–4.8)**	0.67 (0.0068–4.8)	0.292	ns
*TGBF1*	**1.1 (0.54–1.4)**	0.85 (0.33–2.1)	1.1 (0.11–1.7)	> 0.999	ns

‘ns’ indicates ‘not significant’

Since the selected genes were previously found to be preferentially expressed on sorted CD4 T cells while we isolated RNA from whole blood, as opposed to sorted memory CD4 T cells, we further explored correction of the results for the fold change in CD4 counts per mL of blood related to day 0 of ART, to account for the fact that the number of CD4 T cells per mL blood was increasing as a result of successful ART (results summarised in Table 3). It was interesting to find that only the *CD38* gene showed an increased expression relative to day 0 at 1M and 3M of ART, while the *PRF1* gene showed a slight increase relative to day 0 at 1M of ART. At the same time, *ARGHEF18*, *CD27*, *LEF1*, *CCL5*, *FAM129A*, *GZMK* and *RORC* showed a significantly decreased expression (Table 3, p values in bold). Following Bonferroni correction for multiple comparisons however, only the decrease in *CD27* gene expression remained statistically significant.

**Table 3:** Fold change (median and IQR) of gene expression between day 0 and 1M, 3M, 6M of ART, with correction for increasing CD4 counts. Increased gene expression is indicated by values >1. p-values listed compare gene expression between day 0 and 6 months of ART, as well as after Bonferroni correction for multiple comparisons (p_corr_*).

Gene	1M	3M	6M	p value	p_corr_*
**TCM specific genes**					
*ARGHEF18*	0.88 (0.43–1.2)	0.68 (0.3–1.8)	0.52 (0.21–1)	**0.047**	ns
*AXIN2*	0.71 (0.13–1.3)	0.5 (0.15–1)	0.21 (0.085–0.57)	0.121	ns
*CCR7*	0.54 (0.23–1.1)	0.54 (0.21–0.94)	0.28 (0.15–0.72)	0.071	ns
***CD27***	0.77 (0.49–1.6)	0.85 (0.31–1.3)	0.4 (0.16–0.55)	**0.001**	**0.03**
***CD38***	**1.3 (0.62–2)**	**1.1 (0.48–2.6)**	0.81 (0.32–2.5)	0.383	ns
*ICOS*	0.58 (0.32–1.3)	0.81 (0.4–1.3)	0.46 (0.21–1.5)	0.601	ns
*IGF1R*	0.45 (0.31–0.96)	0.39 (0.16–1.7)	0.33 (0.11–0.8)	0.213	ns
*ITK*	0.82 (0.18–2.5)	0.73 (0.29–2.5)	0.53 (0.15–1.6)	0.688	ns
*LEF1*	0.49 (0.27–2)	0.65 (0.15–3.9)	0.3 (0.05–0.67)	**0.040**	ns
*NFKB1*	0.7 (0.28–1.3)	0.77 (0.27–1.4)	0.51 (0.22–0.9)	0.254	ns
*PRKCA*	0.48 (0.12–2.8)	0.2 (0.015–1.8)	0.32 (0.055–3.7)	0.579	ns
*SELL*	0.93 (0.54–1.4)	0.74 (0.49–1.3)	0.83 (0.3–1.3)	0.462	ns
*TCF7L2*	1 (0.36–2.3)	0.77 (0.19–2.9)	0.66 (0.08–2.1)	0.216	ns
**TEM specific genes**					
*CCL5*	0.77 (0.51–1.2)	0.84 (0.42–1.3)	0.45 (0.16–0.81)	**0.007**	ns
*CCR2*	0.47 (0.2–0.84)	0.44 (0.058–1.7)	0.29 (0.073–0.92)	0.351	ns
*CCR4*	0.6 (0.34–1.4)	0.34 (0.075–1.1)	0.33 (0.033–1)	0.056	ns
*CCR5*	0.85 (0.46–1.4)	0.58 (0.14–1.4)	0.29 (0.055–1.6)	0.054	ns
*FAM129A*	0.74 (0.48–1.3)	0.51 (0.34–1.1)	0.48 (0.16–0.65)	**0.004**	ns
*GATA3*	0.52 (0.32–1.2)	0.39 (0.17–0.85)	0.43 (0.06–1.1)	0.350	ns
*GNLY*	0.54 (0.28–0.88)	0.64 (0.36–1.3)	0.35 (0.15–0.82)	0.328	ns
*GZMA*	0.67 (0.39–1.2)	0.66 (0.4–1.2)	0.47 (0.16–1.1)	0.148	ns
*GZMB*	0.76 (0.31–1.2)	0.81 (0.37–2.3)	0.39 (0.11–0.73)	0.064	ns
*GZMK*	0.59 (0.34–1.6)	0.61 (0.25–1.5)	0.33 (0.11–0.67)	**0.016**	ns
*IFNG*	0.6 (0.25–1.4)	0.99 (0.32–1.5)	0.77 (0.05–1.3)	0.446	ns
*IL2RB*	0.68 (0.29–1.1)	0.95 (0.38–1.9)	0.31 (0.033–0.91)	0.066	ns
*PRF1*	**1.1 (0.27–6.3)**	0.67 (0.23–7.1)	0.51 (0.085–2)	0.120	ns
*PRR5L*	0.88 (0.37–1.6)	0.58 (0.18–1.6)	0.75 (0.13–2)	0.351	ns
*RORC*	0.83 (0.3–2.1)	0.39 (0–1.7)	0.09 (0–0.4)	**0.008**	ns
*TBX21*	0.75 (0.26–2.7)	0.69 (0.14–3.5)	0.59 (0.18–2)	0.470	ns
*TGBF1*	0.73 (0.38–1.1)	0.6 (0.3–1.3)	0.67 (0.29–1.2)	0.993	ns

‘ns’ indicates ‘not significant’

**Fig 4:**
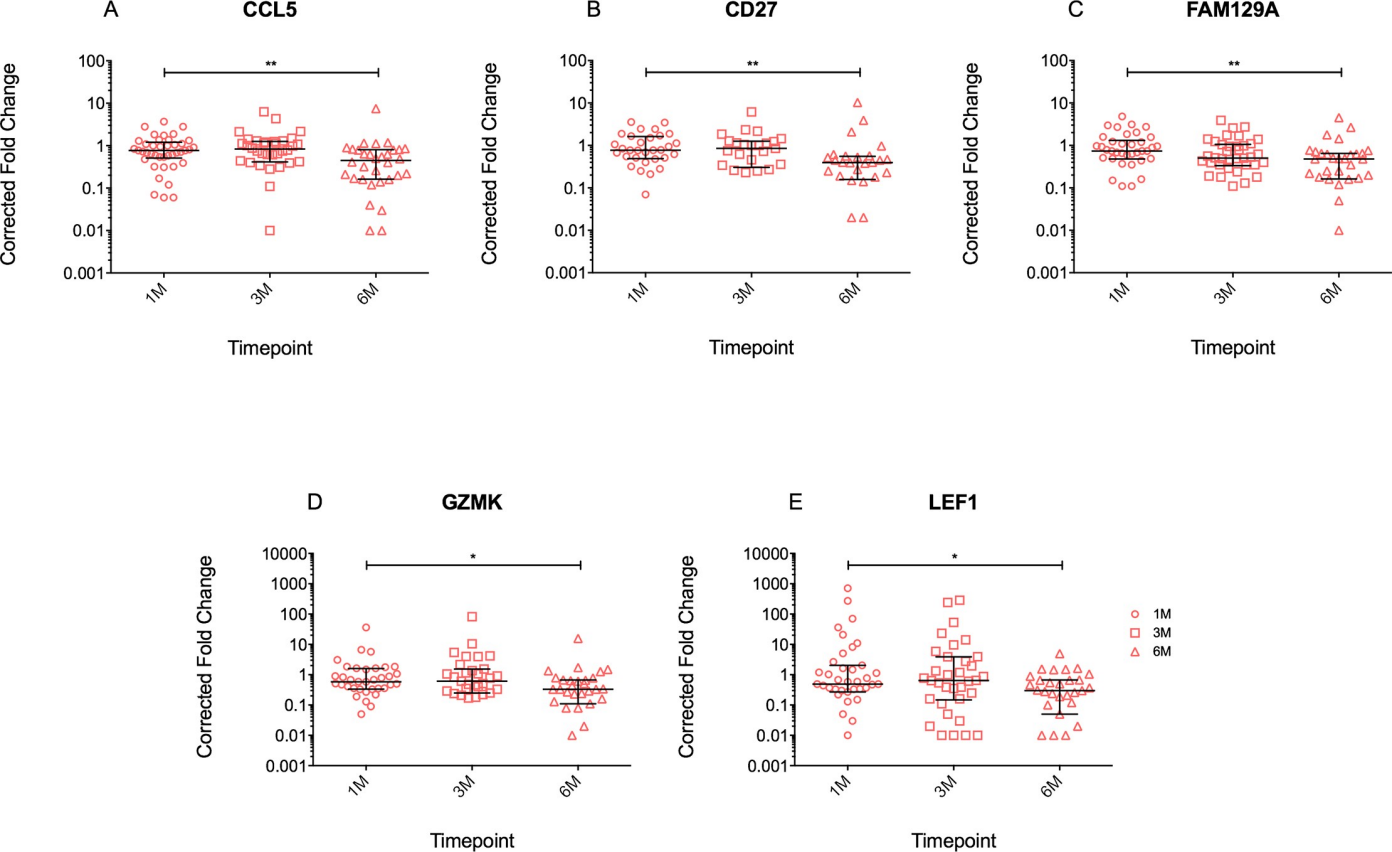
Fold change of gene expression at 1M, 3M and 6M of ART, for the genes that showed significant change over time both with and without correction for increasing CD4 count.

**Fig 5:**
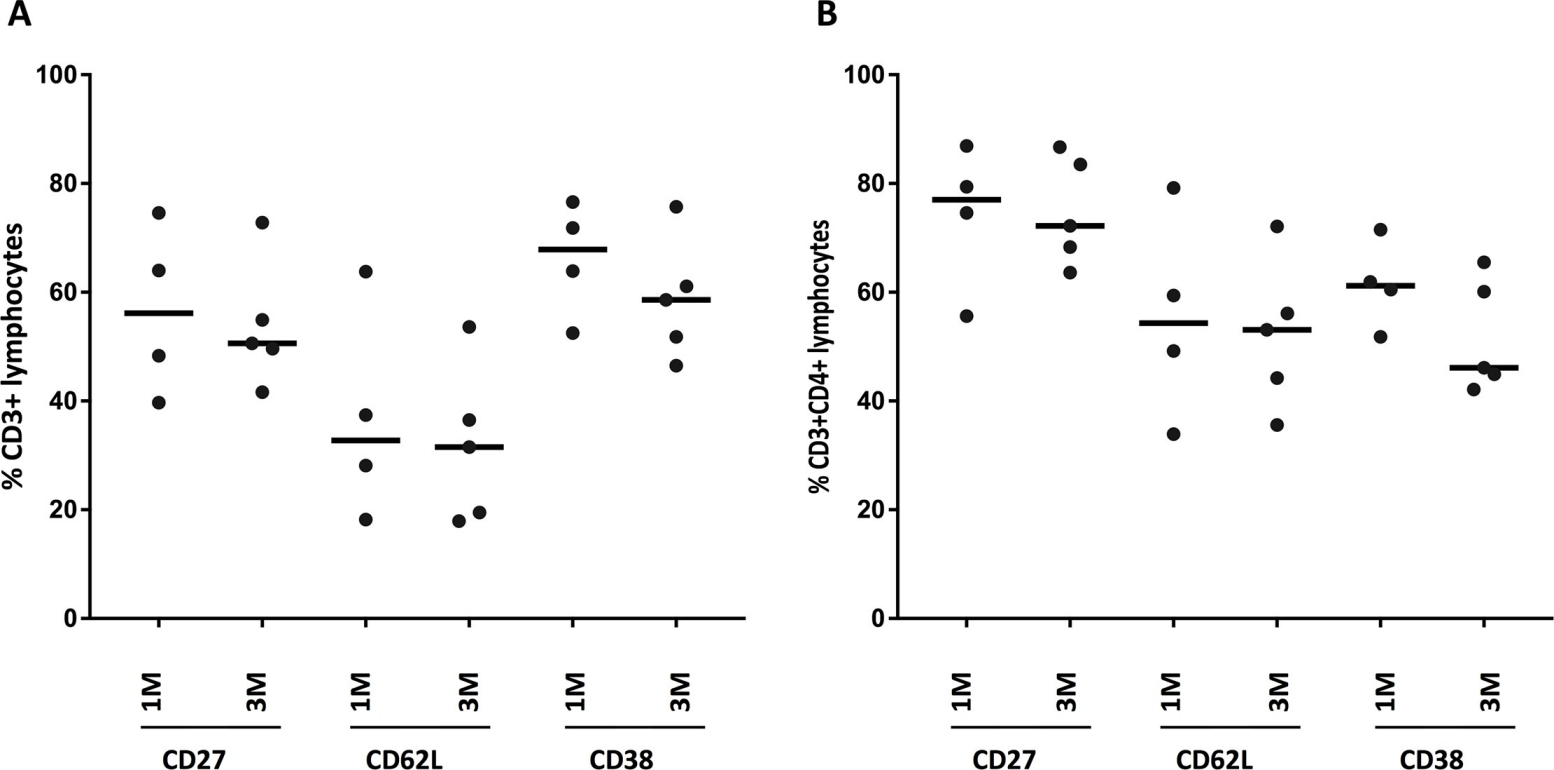
The proportion of (a) CD3+ and (b) CD3+CD4+ lymphocytes expressing CD27, CD62L and CD38 as determined by flow cytometry, using PBMC from a subset of HIV infected patients on ART at various timepoints.

The overlap of genes that showed significant decrease over time both with and without correction for increasing CD4 counts were *CD27*, *LEF1*, *CCL5*, *FAM129A* and *GZMK*, as illustrated in Fig 4. In a subset of patients with available stored PBMC, we performed flow cytometry experiments to assess the surface expression of CD27, CD62L (encoded by SELL) and CD38 on CD3^+^ and CD3^+^CD4^+^ lymphocytes. While this experiment was limited by the number of patients with remaining frozen PBMC and hence the numbers are too small to perform meaningful statistical analyses, the results support a trend in decreasing CD27 at protein level (Fig 5).

## Discussion

In order to identify mechanisms of ART-mediated prevention of TB in HIV-1 infected persons, we investigated the expression of 30 selected genes longitudinally in *Mtb* sensitised persons starting ART, using qRT-PCR. The genes included cytokines and cytokine receptors, chemokine receptors, inhibitory molecules, co-stimulatory molecules, kinases, transcription factors, molecules that play a role in homing and migration and effector molecules. Overall, 13 genes: *ICOS*, *SELL*, *PRKCA*, *TCF7L*, *LEF1*, *NFKB1*, *CD38*, *ITK*, *IGF1R*, *ARHGEF18*, *AXIN2*, *CCR7* and *CD27*, had previously been shown to have a higher expression in sorted *Mtb* specific TCM while 17 genes: *GNLY*, *RORC*, *TGFB*, *PRF1*, *CCL5*, *IFNG*, *CCR2*, *GATA3*, *GZMA*, *GZMB*, *GZMK*, *PRR5L*, *TXB21*, *IL2RB*, *CCR4*, *CCR5* and *FAM129A* had been shown to have a higher expression in sorted *Mtb* specific TEM [10].

The results indicated significantly decreased gene expression for a number of genes at 6 months of ART, reflecting a decrease in immune activation as a result of decreasing HIV viral load. However, following correction for multiple comparisons, only the decrease in *CD27* expression remained statistically significant. CD27 is present on the surface of CD4 T cells and is a receptor involved in co-stimulation. CD27 downregulates as naïve T cells differentiate into central memory T cells and subsequently effector memory T cells, thus central memory T cells tend to express the CD27 while effector memory T cells lack the CD27 receptor [14]. CD27 expression on circulating *Mtb*-specific T cells has been shown to discriminate active tuberculosis from latent tuberculosis infection in HIV uninfected adults and children, suggesting that loss of CD27 expression could be due to persistent antigenic stimulation and might relate to increased homing to the disease site [15, 16]. Studies involving HIV-infected persons showed rapid depletion of *Mtb*–specific CD4 T cell responses early after HIV infection [17] and in contrast to HIV-uninfected, found that *Mtb*-specific CD4 T cell populations from HIV+LTBI+ persons were often dominated by CD27-negative cells [18]. CD27 expression has been proposed as a tool for active and latent TB diagnosis in HIV infected persons as well [16, 19], and further studies indicated HLA-DR expression on *Mtb*-specific CD4 T cells to be promising and comparable [20] ([21]. The impact of ART on the restoration of antigen-specific T cells was shown to be dependent on the co-pathogen specific CD4 T cell memory phenotype, with differences between *Mtb*-specific and CMV-specific memory CD4 cell reconstitution [9, 22]. However, the definition of the memory CD4 T cell is based on the expression of surface markers and a decrease in CD27 transcript detected in whole blood, as shown by our data, cannot extrapolate to the restoration of memory CD4 T cells during ART, necessitating further studies based on sorted memory cells.

We also found increased gene expression at one month of ART for a number of genes, with several showing an increase at three months as well. Only *CD38* and *SELL* showed an increase at all three timepoints studied. Human CD38 is the mammalian prototype of a family of proteins which share structural similarities and an ectoenzymatic activity involved in the production of calcium mobilizing compounds. CD38 is found on the surface of many immune cells and plays a role in the regulation of intracellular calcium, that in turn controls numerous processes such as cell activation and proliferation, hormone secretion and immune responses. Besides the enzymatic activity, the molecule performs as a receptor, responsible for adhesion and signalling in leukocytes [23]. SELL is the gene responsible for the transcription of L-selectin, also known as, CD62L. The protein product of SELL is a homing receptor required by lymphocytes to enter secondary lymphoid tissues. CD62L is expressed on the surfaces of lymphocytes and granulocytes, with central memory T cells expressing L-selectin to localize in secondary lymphoid organs, and effector memory T cells not expressing CD62L [24].

We further explored the data with respect to the ART induced expansion of CD4 T cells, by calculating the fold change corrected for the fold increase in CD4 counts. However, given the broad cellular distribution of many of the molecules studied (such as CCR7, LEF, TCF1, CD27, and CD62L being very abundant on CD8 T cells and other cells, while CD38 is even more highly expressed on T cells other than CD4 T cells), normalization may not have been necessary. Nevertheless, we thought it would be informative to see if changes to these genes during ART can also be detected after normalization to CD4. It was interesting to see that the number of genes showing an increased expression was more restricted, with only CD38 being increased at 1M and 3M of ART, while PRF1 slightly increased at 1M of ART.

The activation of reference genes according to experimental and / or biological conditions is an important factor to consider in gene expression studies, as reviewed [13, 25]. Additionally, *18S* is more stable for T cell specific genes, while *ACTB* and *GAPDH* can be more variable upon activation [25]. Considering that the genes we evaluated, while CD4 T cell specific, could also be expressed by other T cell subsets, and that their expression could potentially be affected by ART induced immune de-activation (in line with decreasing viral load on ART), we elected to use the average expression of the three reference genes for the analysis.

Our study has several limitations that preclude any conclusion on the regulation of memory CD4 T cells during ART: (1) the 30 genes included for analysis were identified in 7 HIV uninfected persons, while our results are based on samples from HIV infected persons during ART. Thus, HIV infection in itself might potentially affect gene expression. (2) We did not have RNA from an HIV uninfected control group. (3) We extracted RNA from whole blood as opposed to sorted cell populations and (4) the genes evaluated are also expressed by non-CD4 T cells present in whole blood. Examination of isolated CD4 T cells alone could also have the limitation of altered gene expression as a result of any manipulation involving the isolation of specific T cell subsets, thus altering the gene expression reflected in bulk whole blood analyses. We believe the answer is to perform single cell sequencing on sorted *Mtb* specific CD4 memory T cells and that is our intention in a future study. An additional limitation is that we did not assess the effect of HIV on the gene expression of T cells in patients without ART, however, as current guidelines recommend starting ART immediately after HIV diagnosis, this would not be ethical. Moreover, HIV-1-specific T cells were previously assessed in patients with advanced disease and showed, that while CD4+ T cell numbers increased substantially during the first year of ART, the population did not normalize and the increases were largely due to the expansion of tissue-derived CCR4+ central memory CD4 T cells [26]. A strength of our study is using three different reference genes, and while the original set of 30 genes were determined in relation to beta-2-microglobulin (*B2M*), the potential generalisability of the findings are illustrated by the fact that we used different reference genes.

In summary, we observed a decrease in the expression of a number of the selected genes at various timepoints, illustrating an ART induced decrease in immune activation. In the same time, a number of genes also showed increased expression at various timepoints, suggesting the broad regeneration of the T cell pool in HIV-1 infected adults on ART. Our findings generate hypotheses underlying ART- induced protective immune reconstitution and may pave the way for future studies to evaluate ART mediated prevention of TB in HIV-1 infected persons.

## Supporting information

S1 Table Genes evaluated by RT-PCR using Taqman PDARs. (XLSX)
